# Finite element models of the human shoulder complex: a review of their clinical implications and modelling techniques

**DOI:** 10.1002/cnm.2777

**Published:** 2016-03-22

**Authors:** Manxu Zheng, Zhenmin Zou, Paulo jorge Da silva Bartolo, Chris Peach, Lei Ren

**Affiliations:** ^1^ School of Mechanical, Aerospace and Civil EngineeringUniversity of ManchesterManchesterM13 9PLUK; ^2^ The University Hospital of South Manchester NHS Foundation TrustSouthmoor RoadWythenshaweManchesterM23 9LTUK

**Keywords:** human shoulder complex, biomechanics, finite element, glenohumeral joint, computational modelling, arthroplasty

## Abstract

The human shoulder is a complicated musculoskeletal structure and is a perfect compromise between mobility and stability. The objective of this paper is to provide a thorough review of previous finite element (FE) studies in biomechanics of the human shoulder complex. Those FE studies to investigate shoulder biomechanics have been reviewed according to the physiological and clinical problems addressed: glenohumeral joint stability, rotator cuff tears, joint capsular and labral defects and shoulder arthroplasty. The major findings, limitations, potential clinical applications and modelling techniques of those FE studies are critically discussed. The main challenges faced in order to accurately represent the realistic physiological functions of the shoulder mechanism in FE simulations involve (1) subject‐specific representation of the anisotropic nonhomogeneous material properties of the shoulder tissues in both healthy and pathological conditions; (2) definition of boundary and loading conditions based on individualised physiological data; (3) more comprehensive modelling describing the whole shoulder complex including appropriate three‐dimensional (3D) representation of all major shoulder hard tissues and soft tissues and their delicate interactions; (4) rigorous in vivo experimental validation of FE simulation results. Fully validated shoulder FE models would greatly enhance our understanding of the aetiology of shoulder disorders, and hence facilitate the development of more efficient clinical diagnoses, non‐surgical and surgical treatments, as well as shoulder orthotics and prosthetics. © 2016 The Authors. *International Journal for Numerical Methods in Biomedical Engineering* published by John Wiley & Sons Ltd.

## 1 Introduction

**Figure 1:**
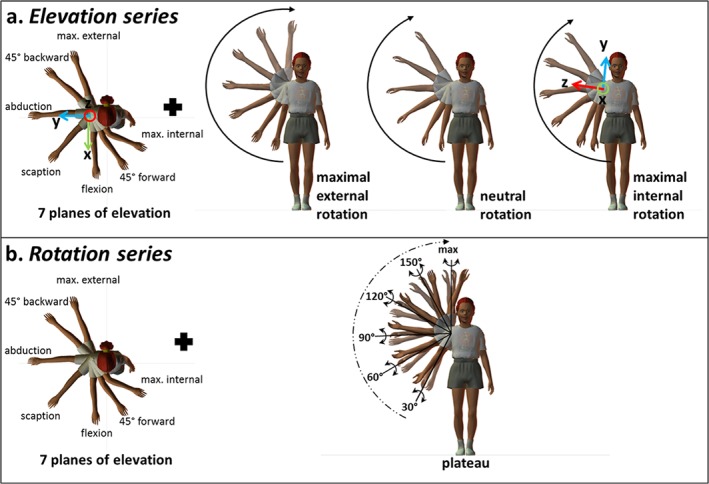
The typical range of motion of the shoulder joint 2.

**Figure 2:**
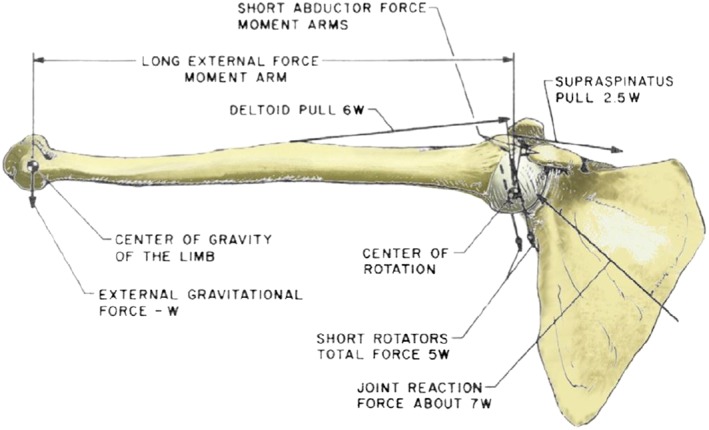
The forces acting at the glenohumeral joint at 90° abduction 5.

The human shoulder is a complicated musculoskeletal structure considered as a perfect compromise between mobility and stability 1. As the major joint in the shoulder complex, the glenohumeral joint permits the greatest range of motion of any joint in the human body. Stability is mainly provided by active muscle actions with a minor contribution from the passive stabilisers, such as glenohumeral capsule, labrum and ligaments etc. The articular surface of the glenoid is considerably smaller than that of the humerus, which facilitates the large range of movement of the joint (see Figure 1 for the typical range of motion of the shoulder joint) 2, 3. In combination with the motion of the scapulothoracic joint, the range of motion of the human upper extremity covers about 65% of a sphere 4. However, on the other hand, the poor congruency of the glenohumeral articular surface challenges joint stability. Translational forces parallel to the articular surface exceeding the stabilising capacity of the joint are the biomechanical reason of joint dislocations. Similar to the other synovial joints, such as the hip, knee or elbow joint, in the glenohumeral joint, those forces have to be counteracted by muscles, ligaments and the joint capsule, which orient the joint contact force towards the articular surface, as the poor articular congruency provides very little additional stability (see the forces of shoulder joint at 90° abduction in Figure 2) 5. This characteristic joint configuration results in high incidences of glenohumeral joint dislocations and most probably predisposes the patient to other painful soft tissue shoulder conditions. However, our understanding of the in vivo biomechanical functioning of the shoulder complex is still very limited. Little is known about the individual contribution of each component of the shoulder musculoskeletal structure to joint stability and mobility and their relationship with each other.

Traditional biomechanical measurements are limited by the existing measuring techniques and ethical issues, and the in vivo internal loading condition of the shoulder musculoskeletal complex is almost unmeasurable 6. Most of the experimental studies to investigate the load transfer in the shoulder structure were limited to in vitro conditions 7, 8, 9, 10, 11. In this scenario, a computational method based on musculoskeletal models provides a valuable tool to estimate the biomechanical behaviour of the shoulder complex under different loading conditions. Computational shoulder models can be roughly classified as two major categories: multi‐body models based on rigid body dynamics and finite element (FE) models based on continuum mechanics. In multi‐body models, the body segments are assumed to be rigid bodies without deformations and muscles are simplified as single line actuators without 3D volume. In combination with muscle wrapping and muscle force estimation methods (optimisation‐based or EMG‐driven), these kinds of models are typically used for determining muscle forces in vivo 12, 13, 14, 15, 16, 17. Through dynamic simulation analysis, multi‐body models have the potential to investigate neuromuscular control strategies, musculoskeletal dynamics and simulated surgical interventions 18. However, because of the major model simplification, the sophisticated deformations, stress distributions and interactions of different components of the shoulder musculoskeletal structure cannot be simulated by using multi‐body models. Those are critical contributors to the in vivo biomechanical and physiological functioning of the shoulder complex and therefore make it difficult to make any clinically useful conclusions from data provided by these methods. Moreover, measurement data used for driving multi‐body models normally suffers from skin artefacts because of skin mounted markers used in motion analysis. Despite those limitations, multi‐body models provide a valuable tool to improve our understanding of the in vivo biomechanical functioning of the musculoskeletal system 19.

On the other hand, continuum mechanics models based on a FE method offer a powerful tool to assess the internal loading conditions of the shoulder musculoskeletal structure 20. They can provide valuable estimates of stress and strain distributions in the bones and soft tissues, which are usually not measurable in vivo. The FE method was first developed to solve elasticity and structural analysis problems in 1940s 21. Its basic concept is the discretisation (division) of complex mechanical structures into finite numbers of separate components with simple geometry called elements. In this way, complex nonlinear problems become solvable numerically. Nowadays, the FE method has been widely used in different engineering fields for system design and analysis 22. Over the past decades, the FE method has also been increasingly used for investigating a large range of problems in biomechanics and orthopaedics 23. According to a recent study, the number of articles using FE analysis in biomechanics appears to be increasing geometrically based on the PubMed database 24. In FE shoulder modelling, the biggest challenge is how to properly represent the complicated structures and materials of the shoulder musculoskeletal system. This paper provides a critical review of the previous studies using FE models to investigate shoulder biomechanics, which are roughly categorised according to the physiological and clinical problems addressed: glenohumeral joint stability, rotator cuff tears, joint capsular and labral defects, and shoulder arthroplasty. The key modelling techniques used in each of those studies are listed in Table I. The articles reviewed in this study were found based on the Web of Science and PubMed databases by using the keywords of ‘FE’, ‘numerical simulation’, ‘glenohumeral joint’ and ‘shoulder joint’.

**Table I:** Key modelling techniques and parameters used in the FE shoulder studies reviewed in this paper.

Clinical issue	Dimension	Geometric acquisition		Model components	Material properties		Boundary conditions	Loading conditions	Validation	Reference
		Bones	Soft tissues		Bones	Soft tissues				
**Joint instability**	3D	In vitro CT scans	In vitro measurement for muscle insertions; Cartilages filling the space between bones	Scapula and humerus; major abduction muscles: deltoid, supraspinatus, infraspinatus, subscapularis	Humerus is rigid; Scapula bone is linear elastic, nonhomogeneous material. E (ρ) = E_0_ (ρ/ρ_0_)^2^, ν = ν_0_, where E_0_ = 15 000, ν_0_ = 0.3 ρ_0_ = 1800.^25^, ^26^	Muscles were assumed exponential hyperelastic, incompressibleW = α exp(β(I_1_ − 3)) − αβ/2(I_2_ − 3)Where α = 0.12MPa, β = 1.0; 27, 28, 29Cartilage was defined based on the Neo‐Hookean incompressible constitutive law.W = C_10_ (I_1_ – 3) with C_10_ = E/4(1 + ν) where C_10_ = 1.79 (E = 10, ν = 0.3). 30	Humerus fixed in transverse plane, vertical translation supported by a spring; Scapula limited by spring elements fixed at spine; Muscles attached fixed with scapula	Initial pre‐stress 1.5 kPa on all muscles; Artificial gradual displacement on infraspinatus (external rotation) and subscapularis (internal rotation)	NO	Büchler P, et al.^31^ (2002)
	3D	Same as 31 Same as 31		Scapula and humerus; major abduction muscles and deltoid	Rigid	Cartilage was defined based on the Neo‐Hookean incompressible constitutive law by W = 1.8(I_1_ − 3); Muscle was modelled using parallel stiff fibres embedded along the principal direction of the muscle volume, which was described by a soft Neo‐Hookean material with W = 0.5(I_1_ − 3).	Estimated muscle forces based on literature data	Artificial rotation of scapula and humerus; A humerus weight of 37.5 N applied on mass centre	Validate against literature^32^ and in vitro study^33^, ^34^	Terrier A, et al.^35^ (2007)
	3D	Literature data		Humerus and scapula; ligaments	Rigid	Cartilages defined as Neo‐Hookean hyperelastic, incompressible material.C_10_ = E/4(1 + ν) D_10_ = E/6(1 − 2 ν) Where E = 10, ν = 0.4 30, 31	Humerus fixed in sagittal plane, unconstrained laterally	Humerus moved 1.2 mm to contact glenoid surface; 50 N compressive load applied on humeral head laterally; 7, 11 Move humerus in anteroinferior direction about 17 mm	Validated against cadaver studies 7, 11	Walia P, et al.^36^ (2013)
Clinical issue	Dimension	Geometric acquisition		Model components	Material properties		Boundary conditions	Loading conditions	Validation	Reference
		Bones	Soft tissues		Bones	Soft tissues				
	3D	In vitro CT scans for scapula Literature data for humerus	Published anatomical data (cartilage and labrum)	Humerus and glenoid; cartilage and labrum	Isotropic linear‐elastic (E = 18 000, ν = 0.35)	Cartilage and labrum are defined same as 31 Cartilage: C_10_ = 1.79 (E = 10, ν = 0.4) and Labrum: C_10_ = 12.5 (E = 70, ν = 0.4).	Estimated active muscle forces from multi‐body model; Artificial glenohumeral elevation in scapular plane from 0° to 80° with arm weight 35 N applied at arm centre of mass		Validated against in vitro^33^, ^37^ and in vivo (EMG)^15^, ^38^ studies	Favre P, et al.^39^ (2012)
**Rotator cuff tears**	2D	In vitro MRI scans		Humerus and glenoid; supraspinatus, supraspinatus tendon	Rigid	Supraspinatus tendon was defined as biphasic, linear, fibre reinforced composite with longitudinally arranged collagen fibres (E = 800) acting with an extrafibrillar matrix (plain stress element with E = 8, ν = 0.497).^40^	Humeral head fixed	Estimated theoretical load and angle on supraspinatus	No	Luo ZP, et al.^41^ (1998)
	2D	In vivo MRI (humeral head)	In vivo MRI for supraspinatus In vitro measurement for cartilages	Humeral head; supraspinatus tendon and cartilages	Humeral head was divided to three regions: cortical bone (E = 13 800, ν = 0.3); subchondral bone (E = 2780, ν = 0.3); cancellous bone (E = 1380, ν = 0.3)^42^	Supraspinatus tendon: E = 168, ν = 0.497. Articular cartilage: E = 35, ν = 0.450.^40^, ^43^ Medium values between the tendon and the cancellous bone were defined for calcified fibrocartilage (E = 976, ν = 0.366) and noncalcified fibrocartilage (E = 572, ν = 0.432).	Humeral head fixed	Estimated theoretical load and angle on supraspinatus	Validated against literature data 41	Wakabayashi I, et al.^32^ (2003)
	2D	Same as 32	Same as 32	Humeral head; supraspinatus tendon and cartilages	Same as 32	Same as 32	Humeral head fixed	Estimated theoretical load and angle on supraspinatus	Validated against literature data 41	Sano H, et al.^44^ (2006)
	3D	In vivo CT scans for humeral head	In vivo MRI for tendon; In vitro measurements for calcified and noncalcified cartilages	Humerus head; supraspinatus tendon and cartilages	Same as 32	Same as 32	Humeral head fixed	Estimated theoretical load and angle on supraspinatus	No	Seki N, et al.^45^ (2008)
Clinical issue	Dimension	Geometric acquisition		Model components	Material properties		Boundary conditions	Loading conditions	Validation	Reference
		Bones	Soft tissues		Bones	Soft tissues				
**Rotator cuff tears**	3D	In vitro CT scans for scapula and humerus	Cryosection photos for glenoid, humeral cartilages and rotator cuff tendons	Scapula and humerus; humeral articular cartilage and rotator cuff tendons	Rigid	Cartilage was modelled as a rigid body with a pressure–overclosure relationship;^46^ Tendons were represented with a linear elastic orthotropic material model (E = 140 ν = 0.497). 41, 47	Scapula fixed; Pre‐defined kinematic boundary for humerus	Artificial rotation from 45° internal to 45° external about an axis parallel to the humeral shaft;	Validated against in vitro study	Adams C, et al.^48^ (2007)
	3D	In vivo CT scans		Scapula and humerus; rotator cuff tendons and deltoid muscle	Solid E = 15 000, ν = 0.3. 35, 48	Muscle and tendons were defined as non‐linear elastic material; Articular cartilage linear elastic E = 15, ν = 0.45.^49^, ^50^	Scapula fixed	Pre‐defined loads on tendons based on cadaver studies 8, 9, 51	Validated against in vitro study^50^	Inoue A, et al.^52^ (2013)
**Capsule and labrum defects**	3D	In vitro CT scans		Scapula and humerus; anterior band of IGHL	Rigid	Anterior band of IGHL was represented using fibre‐reinforced composites. (average material properties from literature)	Measured bone kinematics from the cadaver study		No	Debski R, et al.^53^ (2005)
	3D	In vitro CT scans		Scapula and humerus; joint capsule and rotator cuff tendon	Rigid	IGHL was defined as isotropic hypoelastic. (E = 10.1, ν = 0.4)^54^	Measured bone kinematics from the cadaver study^10^		Validated against in vitro study^54^, ^55^	Ellis B, et al.^56^ (2007)
	3D	In vitro CT scans		Scapula and humerus; capsule, ligaments and cartilages	Rigid	Capsular regions have the same ν = 0.4995 but individual E: Anterior band of IGHL E = 2.05 Posterior band of IGHL E = 3.73 Anterosuperior E = 2.12 Axillary pouch E = 4.92 Posterior E = 2.05	Measured bone kinematics from the cadaver study^57^		Validated against in vitro study	Moore S, et al. 58 (2008)
	3D	Same as 58		Scapula and humerus; capsule and cartilage	Same as 58	Same as 58	Measured bone kinematics from the cadaver study		Validated against in vitro study	Ellis B, et al.^59^ (2010)
	3D	In vitro CT scans		Scapula and humerus; capsule, humeral head cartilage	Rigid	Capsule tissues were represented using an isotropic hyperelastic constitutive model.	Measured bone kinematics from the cadaver study		Validated against specimen‐specific in vitro study	Drury N, et al.^60^ (2011)
Clinical issue	Dimension	Geometric acquisition		Model components	Material properties		Boundary conditions	Loading conditions	Validation	Reference
		Bones	Soft tissues		Bones	Soft tissues				
**Capsule and labrum defects**	3D	In vitro measurements		Glenoid labrum and biceps long head tendon	Glenoid defined as elastic isotropic material (E = 1400)	Labrum and biceps defined as elastic isotropic material (E = 241)	Glenoid fixed	Pre‐defined loads on LHBT from muscle activities from literature^61^	No	Yeh ML, et al.^62^ (2005)
	3D	In vitro CT scans		Glenoid; labrum, cartilages of humeral head and glenoid	Rigid	The humeral cartilage was assumed rigid; Glenoid cartilage was isotropic with properties of E = 1.7, ν = 0.018, ϕ_s_ = 0.25, ρ = 1075; Labrum was transverse isotropic with properties of E_p_ = 0.24, E_θ_ = 22.8, ᵛ_p_ = 0.33, ᵛ_θp_ = 0.10, G _θp_ = 2 ϕ_s_ = 0.75, ρ = 1225 Subscripts define the transverse plane (p) and the circumferential direction (θ) of the labrum.^63^, ^64^, ^65^	Glenoid fixed Humeral cartilages constrained from all rotations and displacement along X‐axis direction (anteroposterior oblique direction).	Move the humeral cartilage 1, 2 and 3 mm in +Y direction (superiorly) 50 N applied in −Z direction (medial oblique direction)	Validated against in vitro study	Gatti C, et al.^66^ (2010)
	3D	In vitro CT scans		Glenoid, glenoid labrum, humeral head and glenoid cartilage.	Rigid	Estimated material coefficients of capsule.^53^, ^67^	Measured humerus motion from in vitro experiment	25 N anterior load applied at 60° of glenohumeral abduction and 0°, 30° and 60° of external rotation	Validated against in vitro study	Drury N, et al. 68 (2011)

E: Young's modulus (MPa); ν: Poison's ratio; ρ: density (kg m^‐3^); I_1_: first invariants of the Cauchy–Green tensor; G: shear modulus (MPa); ϕ_s_: solid volume fraction; W: strain energy density function; C_10_, D_10_: material property constants.

## 2 FE Modelling of Shoulder Complex

### 2.1 FE models of glenohumeral joint stability

The low congruity of the articular joint surfaces in the shoulder affords its large range of motion; however, it predisposes it to being the most commonly dislocated joint of the body. A number of studies have been conducted to investigate instability of the glenohumeral joint by using FE models considering the major components of the shoulder complex.

Buchler et al. 31 used a FE glenohumeral joint model, consisting of the major rotator cuff muscles and bones, to investigate the changes in joint contact stresses because of the changes of the shape of the humeral head and the glenoid contact shape and orientation in both healthy and pathological conditions. It was found that the changes in shape of the humeral head because of joint disorders (e.g. osteoarthritis) may reduce joint stability. However, one of the drawbacks of this study is lack of validation. This FE glenohumeral joint model was also used for analysing the biomechanical effect of the shapes of prosthetic humeral heads after shoulder arthroplasty 69. Two prosthetic designs were examined against an intact shoulder: the second‐generation (Neer II) and a patient‐specific anatomical implant. Similar to the previous FE simulation, joint contact stresses (location and magnitude) were calculated and compared in three cases: intact shoulder, Neer II and patient‐specific condition. The result showed that the patient‐specific implant produced closer biomechanical conditions in both stress location and magnitude to the intact shoulder than the second generation implant. The Neer II implant moved the joint contact area eccentrically and led to bone contact stresses being up to 8 times higher than in the intact shoulder.

**Figure 3:**
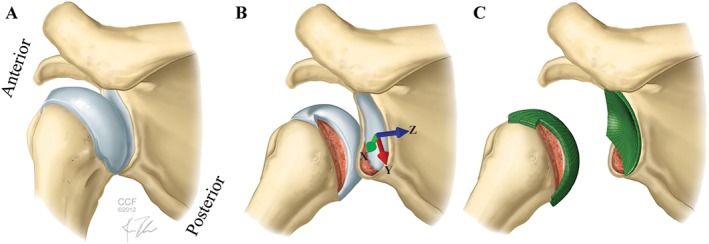
(A) Intact shoulder at 0° abduction; (B) combination of Hill–Sachs and bony Bankart lesion at 90° abduction; (C) FE mesh in combined case 36.

Terrier et al. 35 used a 3D FE model of the shoulder to investigate the biomechanical consequence of supraspinatus deficiency with a major focus on the reduction in glenohumeral joint stability that could lead to secondary osteoarthritis. The effect of supraspinatus deficiency was examined by FE model analyses in both healthy and pathological conditions (considered as a full supraspinatus tear). The result suggested that supraspinatus deficiency increases the upward migration of the humeral head resulting in increased eccentric loading, and thus decreases glenohumeral joint stability. A similar FE shoulder model was used for investigating the effect of combined defects of the humeral head (Hill–Sachs) and of the glenoid (bony Bankart lesion) by Walia et al. (see Figure 3) 36. It was found that the glenohumeral joint stability (defined as the ratio of shear force to compressive force) was decreased from 43% to 0% for the combined presence of both lesions compared to the normal healthy shoulder.

A recent FE model of the glenohumeral joint used estimated muscle forces as the loading condition, and the humerus was allowed to move freely with six degrees of freedom 39. These loading and boundary conditions enable the FE analyses to simulate motions of the shoulder complex closer to its realistic physiological condition than those with pre‐described or artificially defined constraints. The FE analyses used tissue deformations, contact areas and contact pressures to evaluate glenohumeral joint stability. This study provides a useful framework for future FE studies of the shoulder complex. The major limitation of the study is that only the scapula and the humerus bones were considered, and the 3D geometry and structure of muscles and other soft tissues were neglected in the model. Their interactions with the bones and other musculoskeletal components could not be examined in the FE analyses.

In the studies discussed, different methods were used for quantifying glenohumeral joint stability. Buchler et al. 31 used an average of the contact area between the humeral head and the glenoid. Similarly, Terrier et al. 35 calculated the contact point on the glenoid to measure joint stability. Walia et al. 7 used a stability ratio (shear force over compressive force) defined in a cadaveric study. In a recent study by Favre et al. 39, glenohumeral joint stability was defined as the shear force required to dislocate the joint under a 50‐N compressive load. The common feature of these methods is that glenohumeral joint stability is measured as the ability of the joint to keep the humeral head in the centre of the glenoid either through relative displacements or shear and compressive stresses. However, little is known about the relationships between those different methods. There is a lack of comparative studies as well a need to standardise how to quantify and report shoulder joint stability. Moreover, for simplification, most of the previous modelling studies considered only part of the shoulder musculoskeletal complex by neglecting some important factors that may have considerable effect on joint stability, e.g. muscle to muscle and/or muscle to bone contact forces. This leads to a poor understanding of the individual contribution of musculoskeletal components to shoulder stability. Joint stability is an overall performance that requires effective functioning of each part of the musculoskeletal structure 70. Therefore, systematic investigations based on more comprehensive modelling with exchangeable evaluation results are needed for future studies.

### 2.2 FE models of rotator cuff tears

The shoulder complex is actively stabilised by contractions of the rotator cuff muscles. Rotator cuff tendon tears are one of the most common pathologies in the shoulder and the supraspinatus tendon is the most frequently affected. Tears can cause chronic shoulder pain, and may lead to secondary degenerative changes in the shoulder (e.g. cuff tear arthropathy). The aetiology of a rotator cuff tear is multi‐factorial with genetic and environmental factors playing an important role. However, so far, the fundamental mechanism that initiates rotator cuff tears remains unclear. A number of studies have been conducted to explore the underlying biomechanical mechanisms which might cause rotator cuff tears using FE shoulder models.

In 1998, Luo et al. 41 used a simplified 2D FE shoulder model for the first time to investigate the initialisation mechanism of rotator cuff tears by analysing the stress environment in the supraspinatus tendon. The stress distribution was evaluated at the humeroscapular elevation angle of 0°, 30° and 60° respectively and also under two different acromial conditions (with and without subacromial impingement). It was found that the high stress concentration generated by subacromial impingement could initiate a tear. Moreover, the results showed that this tear may occur on the bursal side, the articular side or within the tendon rather than only on the bursal side as the traditional mechanical models suggest. Two further studies based on improved Luo's model were conducted. Wakabayashi et al. 32 applied histological differences at the tendon insertion in their FE model to analyse the stress environment of the supraspinatus tendon. This study showed slightly different result from Luo's study and found that the maximum principle stress of the tendon occurs at the region in contact with the humeral head rather than at the insertion point. Whereas Sano et al. 44 examined the stress distribution in the pathological rotator cuff tendon and revealed potential partial thickness tears at three different locations: on the articular surface, on the bursal surface and in the mid‐substance close to the insertion. It was found that high stress concentration occurs at the articular side of the insertion and the site of tear. The two studies used same modelling method and conditions as Luo's original model, but employed different histological parameters to simulate pathological conditions. Although those studies have improved our understanding of the initiation mechanism of rotator cuff tears, they were limited to 2D condition and lacked experimental validations.

The first 3D FE model of rotator cuff tears was reported by Seki et al. 45 in 2008 to analyse the 3D stress distribution in the supraspinatus tendon. It was found that the maximum stress occurs in the anterior portion of the articular side of the tendon insertion rather than at the tendon contact point with the humeral head as suggested by 2D analyses. This explains the frequent occurrence of rotator cuff tears at this site. This improvement was achieved because of the advantage of the 3D model analysis where the anteroposterior direction was investigated showing that the anterior part of the rotator cuff is not in contact with the superior surface of the humeral head. However, this study only analysed part of the shoulder complex at 0° abduction without experimental validation.

Adams et al. 48 used a 3D FE model of the glenohumeral joint to investigate the effect of morphological changes in the rotator cuff tendons following a tear. The result showed that the moment arms of infraspinatus and teres minor muscles were generally decreased. Consequently, the muscles attached to the torn tendons are required to generate more forces for the same motions, and the overall strength of the shoulder is decreased. This study revealed a potential relationship between shoulder strength reduction and sizes and locations of the rotator cuff tears. The magnitudes and general trends of the calculated moment arms were found in reasonably good agreements with the measured data. A limitation of this study is that tendons were divided along the force bearing direction, which only happens in massive cuff tears transverse to tendon collagen fibrils. Cuff tears along tendon collagen fibrils were neglected in the model analysis.

**Figure 4:**
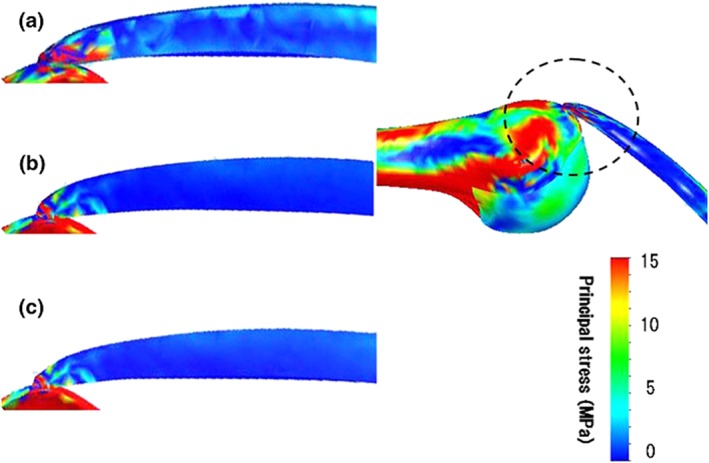
Distributions of tensile stress in the supraspinatus tendon at 90°. View (a), (b) and (c) are anterior, middle and posterior section of the supraspinatus tendon in the sagittal plane respectively 52.

A most recent FE study of rotator cuff tears was conducted by Inoue et al. 52 A 3D FE model including the rotator cuff muscles and the middle fibres of the deltoid muscle was used for investigating the biomechanical mechanism of rotator cuff tears. Different stresses were found in the articular and bursal sides of the supraspinatus tendon resulting in shearing between the two layers, which was believed to initiate partial‐thickness tears (see Figure 4). The limitation of this study is that the muscles and bones were reconstructed based on CT images, which are not very suitable for segmentation of soft tissues. Moreover, the simulated movement was limited to shoulder abduction, and only three rotator cuff muscles and middle fibres of the deltoid were considered in the model.

Those modelling studies investigated the aetiology of rotator cuff tears based on the hypothesis that mechanical stress concentration initiates tendon tissue tear. Although recent studies demonstrated some promising results, the underlying mechanism triggering the pathological process still remains unclear 52. As the shoulder joint is actively stabilised by the rotator cuff muscles, the loading condition at the rotator cuff tendon may have a major effect on the simulation results. However, existing FE models either used in vitro data or were based on roughly estimated tendon force data. Therefore, more accurate in vivo muscle force data is needed in order to provide more convincing results to reveal the fundamental mechanism underlying rotator cuff tears.

### 2.3 FE models of capsular and labral defects

The shoulder articular capsule and labrum are the major passive stabilisers of the glenohumeral joint. The capsule is a thin and loose structure reinforced by surrounding ligaments such as the inferior glenohumeral ligament (IGHL). The labrum is an integral component of the glenoid insertion of the IGHL, which increases the depth and concavity to the glenoid fossa to resist the humeral head translation 60. Injuries, such as a Bankart lesion or HAGL lesion (humeral avulsion of the glenohumeral ligament), are common after an anterior shoulder dislocation. A number of FE studies have been conducted to understand the pathomechanics of the shoulder capsule and labrum. This may lead to new biomechanically oriented strategies to improve clinical diagnosis and surgical interventions to address capsular and labral defects 59.

The first FE study of the capsule was conducted by Debski et al. 53 in 2005, where a FE model of the glenohumeral joint with the anterior band of the IGHL was used for analysing the stress and strain distribution in the IGHL. Although the study revealed the continuous nature of the glenohumeral capsule, the FE model was limited by the fact that only the anterior band of the IGHL was considered and experimental validation was absent. Another FE model of the IGHL was constructed later to examine the strains and forces in the IGHL complex by Ellis et al. 56 It was found through a sensitivity analysis that the predicted strains were highly sensitive to the changes in the ratio of bulk to shear modulus of the IGHL complex. A further study suggested that it is more appropriate to consider the glenohumeral capsule as a sheet of fibrous tissue. This provides useful suggestions on better representation of ligaments in FE modelling 58.

**Figure 5:**
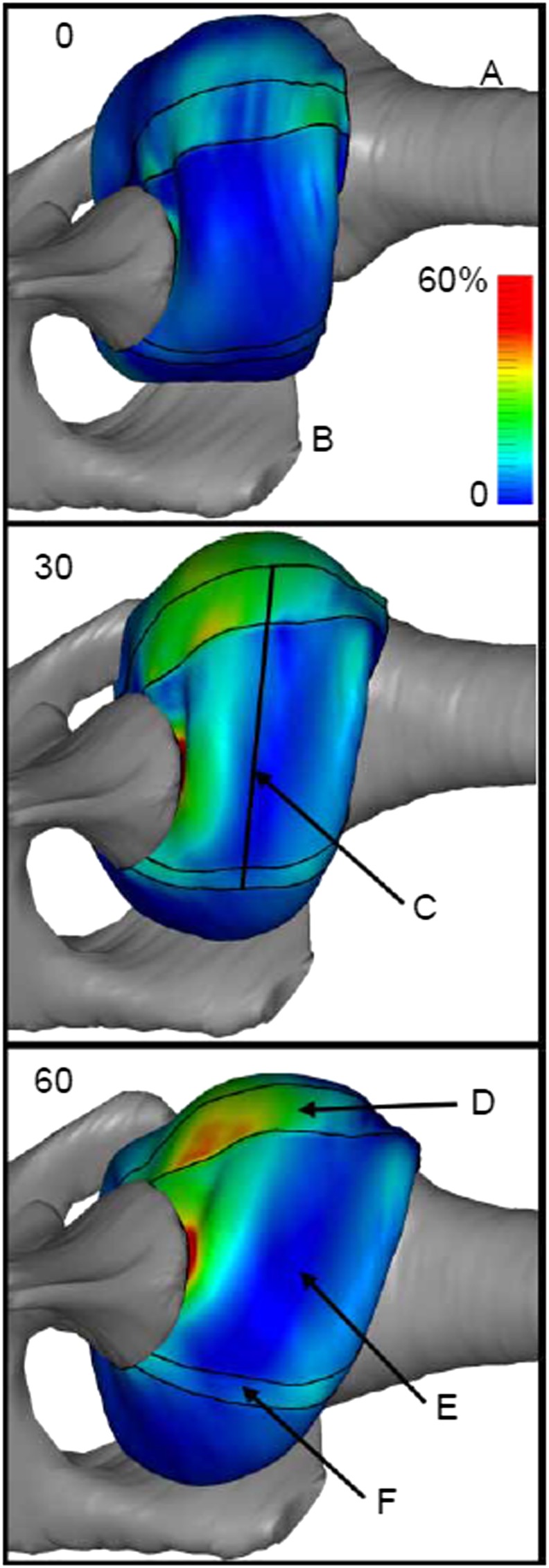
Inferior view of the first principal strain distribution in the left shoulder under 60° of abduction at 0°, 30° and 60° of external rotation. A, Humerus; B, glenoid; C, IGHL mid‐line; D, anterior band of IGHL; E, axillary pouch and F, posterior of IGHL 59.

Recently, Ellis et al. 59 constructed two subject‐specific FE models of the IGHL to analyse the glenohumeral joint positions in clinical examinations by evaluating the strain distribution (see Figure 5). It was suggested that the isolated discrete capsule regions should not be used in analysing the function of the glenohumeral capsule. The study concluded that the positions of 30° and 60° of external rotation can be used for testing the glenoid side of the IGHL during clinical examinations, but are not useful for assessing the humeral side of the IGHL 59. This provides useful suggestions to improvements in clinical evaluation of shoulder instability. The most recent FE study of the glenohumeral capsule was conducted by Drury et al. 68 with a more detailed model construction. The result showed that the glenoid side of the capsule undergoes the greatest deformation at the joint position under 60° abduction and at a mid‐range (20° − 40°) of external rotation. This suggested that standard glenohumeral joint positions could be used for the examination of pathology in the anterior inferior capsule caused by dislocations. FE studies have also been conducted to investigate defects of glenohumeral labrum, such as superior labrum anterior posterior (SLAP) tears, which are normally found among athletes involved in overhead sports 62. Yeh et al. constructed 3D FE models of the superolabral complex with different biceps origins at four orientations during throwing, and the change of peak stress was examined. The maximum stress about 160 Mpa was found in the deceleration phase of throwing, two times of that in the late cocking phase. This high stress in the deceleration phase may lead to tears at the superior glenohumeral labrum. However, no experimental validation was conducted in this study. A later FE study revealed that the superior humeral translation resulting in a shear force to the labrum could be a possible mechanism to the development of SLAP lesions 66.

Recent studies have attempted to consider both the capsule and labrum in FE glenohumeral joint models. Drury et al. 60 constructed a subject‐specific FE model of the glenohumeral joint with the capsule and labrum components to investigate the effect of degenerating tissues on strains in the glenohumeral labrum and capsule by simulating varied labrum thickness and modulus. The results showed that decreasing the thickness of the labrum because of degeneration increases the average and peak strains in the labrum. This increase in strain provides a possible biomechanical mechanism by which the tissue degeneration results in glenohumeral labrum pathology with ageing and also confirms the important, however minor static contribution of the labrum to shoulder stability.

In comparison to the rotator cuff muscles, the capsule and labrum are major passive stabilisers of the shoulder joint. They function to assist with stability when the shoulder joint reaches or exceeds the limit of the joint range of motion. In this scenario, material property might be more important than in vivo loading for investigations of capsule and labrum defects. Although material property studies have been conducted for the capsule ligaments based on specimen‐specific experiments 56, 58, more detailed studies are needed to quantify the complex material behaviour of the capsule and labrum in vivo. Moreover, further studies may need to pay more attention to accurate representation of the glenoid insertion site which varies significantly between subjects 60.

### 2.4 FE models for shoulder arthroplasty

A total shoulder replacement includes the replacement of the humeral head and the glenoid with prostheses that perform as a new joint. Therefore, the prosthesis design is fundamental in shoulder arthroplasty. FE analysis has been widely used in the design of prosthesis especially in investigating the clinically important issue of glenoid component aseptic loosening. The FE studies conducted for shoulder arthroplasty assessment are critically reviewed in this section, and key design parameters and major findings of each study are detailed in Table II.

**Table II:** The fundamental information and major findings of the FE shoulder arthroplasty studies reviewed in this paper.

Implant geometry	Orientation and position	Cement	Prosthesis material	Glenohumeral conformity	Findings	Reference
Keel, stair‐stepped, wedge and screw	N/A	Cemented	Cobalt–chromium metal for backing and polyethylene	N/A	1. An all‐polyethylene implant could provide a more physiological stress distribution for nonaxial loads; 2. A soft tissue layer results in higher stresses; 3. Stair‐stepped and wedge designs demonstrated a more natural stress distribution compared to keel design; 4. Screw orientation does not make much difference.	Friedman RJ, et al.^71^ (1992)
Triangular keel	N/A	Cemented	Cobalt–chromium metal for backing and polyethylene	High and low conformity achieved by varying load distribution over contact area	Fatigue failure could originate from the high stresses in cement.	Lacroix D and Prendergast PJ.^72^ (1997)
Keel	N/A	Cemented all‐polyethylene component Uncemented metal‐backed component		N/A	1. Cemented all‐polyethylene design produced more natural stress overall. 2. Extremely high stress were found in the polyethylene in contact with metal surface	Stone KD, et al.^73^ (1999)
Peg and Keel (DD 1134‐96 and DD 1134‐85 DePuy Inc.)	N/A	Cemented	All‐polyethylene	N/A	A ‘pegged’ anchorage system is superior for normal bone, whereas a ‘keeled’ anchorage system is suitable for rheumatoid arthritis bone.	Lacroix D, et al.^74^ (2000)
Keel	N/A	Cemented and uncemented glenoid component	Polyethylene cup with a metal‐backing	N/A	Uncemented design is a reasonable alternative to fixation with cement.	Gupta S, et al.^75^ (2004)
Peg (Anatomica glenoid component Centerpulse Ltd.)	five component alignments: central, anteverted, retroverted, inferiorly inclined and superiorly inclined.	Cemented	Ultra‐high molecular‐weight polyethylene	N/A	Central alignment is the correct position. Misalignment of the glenoid prosthesis can lead to loosening. Joint replacement depends much on bone stiffness.	Hopkins, AR, et al.^76^ (2004)
Acromion‐fixation	N/A	Cemented	Polyethylene	N/A	High stresses were found in the part of prosthesis that attached to the acromion. The acromion‐fixation design is not a good alternative.	Murphy LA and Prendergast J.^77^ (2005)
Keel	N/A	Cement mantle thickness were examined from 0.5–2 mm	All‐polyethylene	N/A	The cement thinning weakens the cement, whereas the cement thickening makes the implant rigid, consequently increases the stress on bone cement interface. Optimal cement thickness is found to be between 1.0 and 1.5 mm.	Terrier A, et al.^78^ (2005)
Keel	Humeral and glenoid components were implanted based on the manufacturer's recommendation	Cemented	All‐polyethylene	Different values of conformity were tested (1–15 mm of radial mismatch)	Conformity had no influence at 0° of retroversion, whereas, at 15° of retroversion, the contact pressure, cement stress, shear stress and micro motions at bone–cement interface increased by more than 200% and exceeded critical values above 10 mm.	Terrier A, et al.^79^ (2006)
Reversed and anatomical (Aequalis prosthesis, Tornier Inc)	N/A	N/A	All‐polyethylene	N/A	Volumetric wear for the anatomical prosthesis and reversed version were 8.4 mm and 44.6 mm respectively. Contact pressure for the anatomical prosthesis was about 20 times lower than that of the reversed. Anatomical prosthesis showed the consistency with the biomechanical and clinical data.	Terrier A, et al.^80^ (2009)
Four anatomical and one reversed	N/A	Three anatomical models were Cemented One anatomical was Cementless	Three cemented anatomical model were all‐polyethylene; One cementless anatomical model was metal‐back; Reversed model was all metal	N/A	Cementless, metal‐back components are more likely to have stress shielding than cemented all‐polyethylene components regardless of bone quality. The all‐polyethylene components are better in treatment of the shoulder joint.	Quental C, et al.^81^ (2014)

The scapula is connected to the axial skeleton via the clavicle. It provides a mobile yet stable base for humeral movement. The scapula also provides the insertion for a number of shoulder muscles and ligaments. Several studies have been conducted to investigate the biomechanics of the scapula because bone remodelling of the scapula is considered as the first step towards the study of complications of shoulder arthroplasty 82. According to a recent study, A typical bone modelling procedure for creating a patient‐specific FE model involves four steps: (1) geometry acquisition using medical images (CT/MR); (2) segmentation of those images and creating FE meshes; (3) definition of patient‐specific material properties and (4) application of patient‐specific multi‐body loading and boundary conditions. The authors also examined the effect of three major modelling uncertainties, including bone density, musculoskeletal loads and material mapping relationship, on the predicted strain distribution. It was found that the number of uncertain components and the level of uncertainties determine the uncertainty of the results. The limitation of this study is that the material mapping relationship was determined based on cadaveric experiments conducted in vitro rather than from data measured in vivo 83.

Glenoid component loosening is a critical clinical issue in total shoulder arthroplasty. Most of the FE studies of shoulder arthroplasty were designed to investigate this problem. Through FE simulation analyses, the biomechanical effects of different key design parameters, such as implant shape, positioning and orientation, prosthesis material, use of bone cement and articular conformity, were examined. A large number of those studies investigated the effect of the shape of the glenoid component. Different glenoid shapes: keel, stair‐stepped, wedge and screw, were compared in an early study 71. It suggested that the stair‐stepped and wedge designs provided a more natural stress distribution compared to the keel design. However, this study was only limited to 2D condition. A later FE study found that the peg design is superior for normal bones, whereas the keel design is more suitable for rheumatoid bone 74. Based on a failure model, the FE simulation predicted 94% and 86% of bone cement survival probability for the peg design for normal bone and rheumatoid bone conditions respectively. Whereas, the survival probabilities for the keel design are 68% and 99% respectively. Another study has concluded that a novel design with acromial fixation point for the glenoid component is unsuitable for shoulder arthroplasty because of the high stress resulted in the part of prosthesis attached to the acromion 77.

The conformity of the glenoid component with the humerus component has been investigated using FE analyses as well. It was found that a higher conformity has the advantage of moderating cement stress 72, and the effect of conformity is sensitive to the shoulder joint position 79. For example, for the same conformity, the contact pressure, cement stress, shear stress and micro‐motions at the bone–cement interface were increased by more than 200% at 15° of retroversion compared to those at 0° of retroversion. Another FE study showed that the central alignment with the humerus component is the correct position for the glenoid component, and misalignment may lead to glenoid loosening 76.

**Figure 6:**
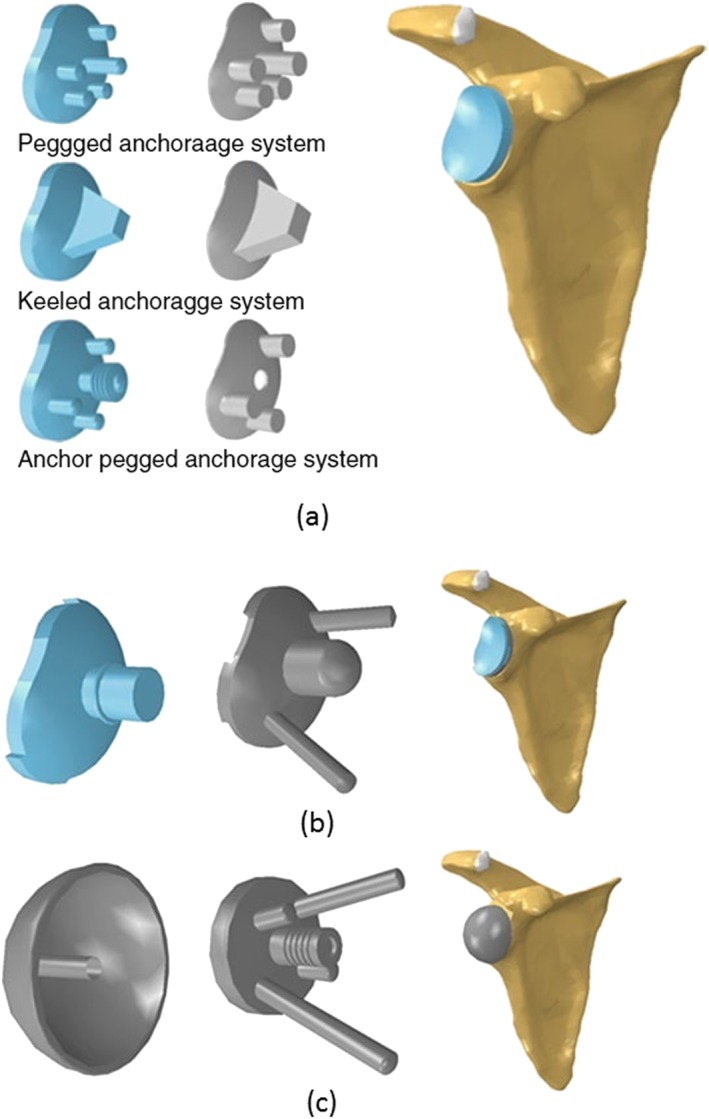
(a) Three anatomical models of the cemented glenoid components (on the left) and their corresponding cement mantles (in the middle); (b) the cementless anatomical model of glenoid component; (c) the reversed glenoid component 81.

Studies looking at the choice of prosthetic components did suggest that metal backed glenoid component might be best 71, 72, 73. However, a recent study has shown that all‐polyethylene cemented glenoid components are likely to be more resilient to aseptic loosening (see Figure 6) 81. The use of bone cement is a controversial element to glenoid component implantation. A number of studies have been conducted to investigate this issue. An early 2D FE study analysed local stresses at the bone implant interface between two glenoid designs. This showed that the cemented all‐polyethylene design produced more natural stress overall although extremely high stresses were found in the interface between the polyethylene and the metal 73. A recent FE study using an integrated model suggested that the cementless, metal‐back components are more likely to have stress shielding than the cemented all‐polyethylene components regardless of bone quality 81. Another study investigating the effect of cement thickness concluded that although a thin cement mantle weakens the cement, a thick mantle makes the implant rigid and consequently increases the stress in the bone–cement interface 78. The optimal cement thickness was found to be between 1.0 and 1.5 mm 78.

The previous studies of total shoulder arthroplasty using FE simulation analyses have greatly improved our understanding of the biomechanical effects of the key design parameters of shoulder implants, e.g. 3D shape, position and orientation, material and articular conformity etc. Future work should involve investigations of some unsolved problems, e.g. the mechanical degradation of the interfaces in cemented components 81, and bone loss in aseptic in both cemented and cementless components 81 as well as glenoid notching in reverse total shoulder arthroplasty. However, similar to the studies investigating shoulder joint instability, the major limitation here is still lack of detailed representation of all major shoulder musculoskeletal components and also physiologically realistic in vivo loading and boundary conditions.

## 3 FE Shoulder Modelling Techniques

For the FE studies reviewed in the preceding section, the key modelling techniques used in each shoulder FE model were listed in Table I. To reduce the computational load, most of those FE models only considered part of the shoulder structure rather than modelling the whole shoulder complex. Therefore, major model simplifications and assumptions were normally involved in the modelling processes. Those simplifications and assumptions could greatly facilitate the computations when representing a complex musculoskeletal structure such as the human shoulder complex. However, this will inevitably lead to discrepancies between the simulated results and the realistic physiological functions. The limitations of those FE models are discussed in this section in terms of the key modelling techniques used: geometric data acquisition, material property representation, boundary and loading condition definition and experimental validation.

Normally, the first step in FE shoulder modelling is to reconstruct the 2D or 3D geometry of hard tissues and soft tissues of the musculoskeletal structure. Different approaches have been used for acquiring the dataset for the geometric construction varying from using literature data 71, average measured data 36 to subject‐specific medical imaging data 31, 32, 39, 41, 44, 45, 48, 52, 53, 56, 58, 59, 60, 62, 66, 68, 84. For geometric construction of bones, the most widely used approach is based on computed tomography (CT) imaging data, for example, the CT image databases for the modelling of the cervical spine and hip joint in our previous studies 85, 86. While in some studies the bone geometry was measured directly in cadaveric dissections or used the datasets from literature 32, 39, 48, 56. For geometric modelling of soft tissues, such as musculotendinous units and ligaments, datasets obtained from magnetic resonance (MR) imaging or colour cryosections were normally used 32, 41, 44, 45, 48. However, the geometric reconstruction of articular cartilages still remains challenging. Assumptions are normally used for defining the geometry of articular cartilage, for example using the estimated average thickness for the whole bone surface 82 or filling the space between the humerus and the scapula with hyaline cartilage 31. Some recent studies used the published anatomical datasets to determine the cartilage geometry 36, 39. Subject‐specific accurate geometric modelling of cartilage will assist a better understanding of the in vivo cartilage mechanics in the glenohumeral joint. In many studies, the geometric data was obtained in vitro. However, to investigate the in vivo functioning of the shoulder complex, the geometric data of the shoulder tissues is acquired preferentially in vivo under unloaded conditions.

Because of the great complexity in the mechanical behaviours of biological materials, it has been proven very challenging to represent the realistic material properties of the shoulder tissues in FE modelling. Major assumptions were typically used in most of the FE shoulder studies. In most cases, bones were assumed to be rigid or isotropic linear elastic material with relatively large Young's modulus, because the deformation of bones is almost negligible compared to that of soft tissues. Muscles and tendons were modelled as isotropic linear elastic materials in many early studies 32, 35, 41, 44, 45, 48. Some later FE studies considered the non‐linearity in the material properties of muscles and tendons which is more accurate and closer to in vivo condition 52. However, only the passive behaviour of the musculotendinous units was represented in the modelling. A most recent shoulder FE study described the material property of muscles by using a constitutive relationship representing both the active and passive behaviour of muscles along the direction of muscle fibres 84, 87. Conversely, tendons were modelled using different parameters to describe their along‐fibre and cross‐fibre properties 84. These detailed properties have been shown to agree with in vivo measurement of the biceps brachii 88. In most cases, joint capsules were modelled as isotropic hyperelastic material 53, 56, and detailed parameters for each region were assigned in some studies which was validated through experimental strain measurements 58. The labrum was normally assumed to be an isotropic material 62. Lately, detailed transverse isotropic material properties were applied in some recent studies which compared well to the experiment measurements 66. For articular cartilages, they were modelled as homogeneous linear elastic material in most of the studies 32, 35, 44, 45, 52, 82. However, in some cases, they were considered to be rigid same as bones by assuming that the material properties of cartilages do not have significant effect on simulation results 48, 66. Biological materials normally have complicated mechanical property, which is anisotropic and nonhomogeneous in nature with nonlinear and viscoelastic behaviours. However, depending on the research problem to be addressed, this material property might be simplified in some cases without compromising the quality of the analysis results. More sensitivity analysis studies are needed in the future to better understand the effect of different material properties 24.

The definition of boundary and loading conditions in previous shoulder FE studies varies dramatically because of the complexity in the joint motions and musculoskeletal loads of the shoulder complex. Many studies only considered part of the complex, and modelled the neglected parts using artificially imposed boundary or loading conditions 31, 32, 41, 44, 45, 62. A number of studies defined their boundary and loading conditions according to the apparatus settings in the cadaveric experiments to facilitate model validations 39, 48, 52, 53, 58, 66, 68, 89. Whereas, in some other studies, boundary conditions were defined by imposing artificially prescribed displacements or rotations of certain muscles or bones 56, 59, 60, 84. However, none of the previously stated boundary and loading conditions are capable of describing the realistic physiological conditions of the shoulder complex where significant muscle activations are involved. Some studies have addressed this and have attempted to use previously determined muscle forces from multi‐body models as boundary and loading conditions 35, 78, 82. But, they either considered the scapula in isolation 78, 82 or performed a 2D analysis only 35. The most appropriate implementation of boundary and loading conditions in shoulder FE modelling is to describe the natural shoulder joint and bone motions driven by physiologically realistic muscle forces without any artificial constraints imposed 39. This kind of physiological boundary condition has been proven to be beneficial in FE modelling of the femur 90. In addition, the FE simulation analyses in most of the studies were only limited to the static or quasi‐static condition. We have applied dynamic FE simulation analysis to study cervical spine and foot biomechanics 86, 91, and have an ongoing study to use dynamic FE analysis to investigate the in vivo functioning of the shoulder complex. Physiologically more realistic loading and boundary conditions are likely to provide the best way to predict the tissue stress environment in vivo.

Experimental validation of FE models is essential because model simplifications and assumptions are normally employed in shoulder FE studies. Unfortunately, some of the studies were not validated or only simply compared to previously published results 32, 41, 44, 45, 71. For in vitro measurement based studies, it is straightforward to validate the models against the specimen‐specific experimental data 36, 48, 52, 58, 66, for example strain gauge data for surface strain 75, 89, 92. However, those validations were conducted based on the data collected in vitro, rather than in vivo data describing the physiological functioning of the shoulder complex. In vivo validations are challenging because of the limitation of current measuring techniques. Theoretically, FE simulation results could be validated in vivo against measured translational/rotational displacement data, strain/deformation data and stress/pressure data. The positions and orientations of bones or joints captured from motion analysis systems or X‐ray systems could provide useful datasets to validate FE simulations in vivo. Recently, we have successfully used this method to validate a FE model of cervical spine 86. Advances in medical imaging domains (e.g. dynamic MR/CT scanning or ultrasound) 93, 94 and force and pressure sensing techniques 95, 96 provide promising methods to validate FE simulation results in vivo against tissue deformation and contact pressure data.

It is evident that more comprehensive musculoskeletal FE modelling with physiologically realistic loading and boundary conditions is needed to further improve our understanding of the in vivo functioning of the shoulder complex. To further this, subject‐specific modelling is the most promising simulation solution. In musculoskeletal modelling, there are normally a large number of uncertainties involved in different components of the system, which is further confounded by inter‐subject variations 24, 97. To rigorously validate the modelling results in vivo, personalised datasets and parameters are desirable. Subject‐specific modelling studies have been successfully applied to other musculoskeletal complexes, such as pelvis 98 and femur 99, but very few to the shoulder joint. Future orthopaedic interventions and surgeries are likely to benefit from patient‐specific biomechanical analyses and assessments before and/or after treatments. This approach has been successfully demonstrated in the total knee arthroplasty 100, 101 and periacetabular osteotomy surgery 85.

## 4 Conclusion

Previous FE studies to investigate shoulder biomechanics have been critically reviewed according to the clinical issues addressed. This confirms that FE modelling is a valuable tool to examine both physiological functions and clinical problems of the shoulder complex. Most of those modelling studies have improved our understanding of the biomechanical functioning of the shoulder joint. Specifically, they normally have one or more research focuses: biomechanical mechanism underlying joint motion, aetiology of joint pathology, clinical diagnoses of joint diseases and shoulder prosthetics design. It may be difficult to say which is the best model, but the most recent models using latest techniques tend to have more complicated configurations and provide more detailed databases than the models before. It is noticeable that more comprehensive model is needed to better understand the in vivo functioning and also the interaction of different components of the shoulder joint. Recently there has been a tendency in shoulder FE studies to construct subject‐specific or patient‐specific FE models informed by muscle force and/or bone motion data from measurement based multi‐body modelling. Despite the aforementioned limitations of multi‐body modelling, this integration offers some promising solutions by providing more accurate boundary and loading conditions.

Fully validated shoulder FE models will greatly enhance our understanding of the fundamental mechanisms underlying shoulder mobility and stability, and the aetiology of shoulder disorders, and hence facilitate the development of more efficient clinical examinations and diagnoses, non‐surgical and surgical interventions and treatments including prostheses. In author's opinion, the model validation may need to be conducted by interactively working with clinicians. First, FE models could be carefully validated for healthy people by using the latest medical imaging and sensing techniques. Then, with some confidences built up, the models could be applied to individual clinical case by using the patient‐specific database provided by clinicians. The models could be further improved based on prediction results and also feedbacks of doctors. Indeed, we still face many challenging problems before the realistic physiological functions of the shoulder mechanism can be accurately represented and analysed in FE simulations.

Future works and challenges involve:

1. Subject‐specific representation of the non‐linear anisotropic nonhomogeneous material properties of the shoulder tissues in both healthy and pathological conditions, and also definition of boundary and loading conditions based on individualised physiological data. Special attention should be paid to the consistency between the FE models and the multi‐body models used for muscle force estimation.
2. More comprehensive models describing the whole shoulder complex including appropriate 3D representations of all major shoulder hard tissues and soft tissues and their delicate interactions, are highly demanded to better understand the biomechanical functioning of the shoulder mechanism.
3. Dynamic FE simulations based on physiologically realistic boundary and loading conditions to better understand the in vivo biomechanical functioning of the shoulder joint during our daily activities.
4. Advanced medical imaging, sensing techniques to quantify in vivo strain and stress distributions of soft and hard tissues in both normal and pathological conditions so as to validate FE models more rigorously.
